# Direct Transformation of Crystalline MoO_3_ into Few-Layers MoS_2_

**DOI:** 10.3390/ma13102293

**Published:** 2020-05-15

**Authors:** Felix Carrascoso, Gabriel Sánchez-Santolino, Chun-wei Hsu, Norbert M. Nemes, Almudena Torres-Pardo, Patricia Gant, Federico J. Mompeán, Kourosh Kalantar-zadeh, José A. Alonso, Mar García-Hernández, Riccardo Frisenda, Andres Castellanos-Gomez

**Affiliations:** 1 Materials Science Factory, Instituto de Ciencia de Materiales de Madrid (ICMM-CSIC), E-28049 Madrid, Spain; gsanchezsantolino@ucm.es (G.S.-S.); chunwei.hsu@mail.mcgill.ca (C.-w.H.); patricia.gant@csic.es (P.G.); federico.mompean@csic.es (F.J.M.); ja.alonso@icmm.csic.es (J.A.A.); marmar@icmm.csic.es (M.G.-H.); riccardo.frisenda@csic.es (R.F.); 2 Kavli Institute of Nanoscience, Delft University of Technology, 2600 GA Delft, The Netherlands; 3 Departamento de Física de Materiales, Universidad Complutense de Madrid, E-28040 Madrid, Spain; nmnemes@fis.ucm.es; 4 Departamento de Química Inorgánica, Facultad de Químicas, Universidad Complutense, E-28040 Madrid, Spain; atorresp@ucm.es; 5 School of Chemical Engineering, University of New South Wales, Kensington, NSW 2052, Australia; k.kalantar-zadeh@unsw.edu.au

**Keywords:** 2D materials, molybdenum trioxide (MoO_3_), molybdenum disulfide (MoS_2_), synthesis, sulfuration

## Abstract

We fabricated large-area atomically thin MoS_2_ layers through the direct transformation of crystalline molybdenum trioxide (MoO_3_) by sulfurization at relatively low temperatures. The obtained MoS_2_ sheets are polycrystalline (~10–20 nm single-crystal domain size) with areas of up to 300 × 300 µm^2^, 2–4 layers in thickness and show a marked p-type behavior. The synthesized films are characterized by a combination of complementary techniques: Raman spectroscopy, X-ray diffraction, transmission electron microscopy and electronic transport measurements.

## 1. Introduction

Two-dimensional (2D) transition metal dichalcogenides (TMDCs) have recently gained interest among the scientific community to solve the weakness of the lack of a bandgap in graphene, which limits its applications in field-effect transistors and digital integrated circuits [1]. The TMDC molybdenum disulphide (MoS_2_) was the first 2D material with an intrinsic bandgap that was isolated [2] and it consists of S-Mo-S layers that are held by weak van der Waal forces in a trigonal prismatic structure [3,4,5,6]. In its bulk form, this material displays an indirect bandgap of about 1.2 eV; nevertheless, it becomes a direct bandgap semiconductor (1.8 eV) when it is thinned down to a monolayer [7]. In addition, when a single-layer MoS_2_ is used as the channel in a field-effect transistor, it exhibits high in-plane mobility and a large current ON/OFF ratio [8]. These are the reasons why molybdenum disulphide has attracted interest for electronic and optoelectronics applications [8,9,10]. Furthermore, it is an attractive candidate for energy conversion [11,12] and storage [13,14], hydrogen evolution reactions [15,16,17] or oxygen reduction reactions [18].

The first methods that were reported for the synthesis of 2D MoS_2_ consisted of mechanical and chemical exfoliation from bulk crystals [2,19,20,21] and, in fact, a lot of studies still use these methods since they provide high-quality single layers. However, these techniques present some problems, like randomly deposited flakes, relatively small coverage area of material and poor control over thickness. A solution for these issues is critical to achieve real-life electronic devices based on MoS_2_ and, therefore, synthesis of large-area MoS_2_ films is a very active research area. The most-explored methods to synthesize large-area MoS_2_ thin films are chemical vapour deposition (CVD) [22,23] and the sulfuration of sputtered molybdenum thin films [24,25,26].

Here, we explore an alternative route to obtain atomically thin MoS_2_ layers: the direct transformation of crystalline molybdenum trioxide (MoO_3_) layers into MoS_2_ nanosheets by sulfurization at moderate temperatures. Up to now, the sulfurization of crystalline MoO_3_ has only been demonstrated to produce MoS_2_ fullerenes and nanotubes, but, here, we demonstrate that it can be also employed to fabricate large-area MoS_2_ layers [27,28]. We characterized the resulting layers by Raman spectroscopy, X-ray diffraction and transmission electron microscopy, finding that the resulting layers showed all the characteristics of polycrystalline MoS_2_. We transferred the as-synthesized films to pre-patterned electrodes to fabricate electronic devices, and we found that they were strongly p-doped, which can be an interesting feature to complement the marked n-doping of mechanically exfoliated or CVD-grown MoS_2_. Our synthesis method does not require a tube furnace with flow gas control as the sulfurization is carried out in a sealed ampoule, simplifying considerably its implementation and reducing its cost.

## 2. Materials and Methods

**Figure 1:**
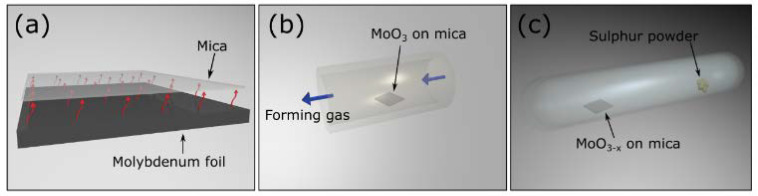
Cartoon of the process followed for the MoO_3_ conversion into MoS_2_. (**a**) MoO_3_ sublimes from a hot molybdenum foil (540 °C) and crystallizes onto a mica substrate. (**b**) MoO_3−x_ is formed after placing the MoO_3_ in a tube furnace at 300 °C in a forming gas atmosphere for 24 h. (**c**) The sulfuration process is performed in a closed glass ampoule at 500–600 °C.

The crystalline MoO_3_ source is obtained by heating up a molybdenum foil (99.99% purity) to 540 °C in air using a laboratory hot plate. At this temperature, the MoO_3_ starts to sublime. A mica substrate is placed above the hot molybdenum foil. The MoO_3_ gas sublimed from the hot molybdenum foil crystalizes on the slightly cooler mica substrate placed on top, as we show in Figure 1a. As reported by Molina-Mendoza et al. [27], this method produces continuous crystalline thin films through a van der Waals epitaxy process thanks to the van der Waals interaction with the mica surface. Note that in the van der Waals epitaxy process there is no need for lattice matching between the substrate and the grown MoO_3_ overlayer.

**Figure 2:**
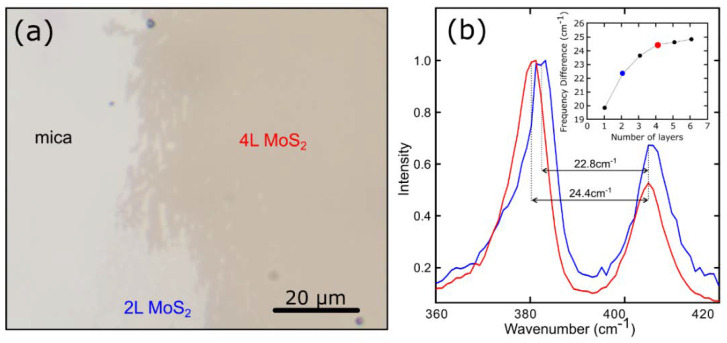
(**a**) Optical image of a large-area MoS_2_ on a mica substrate. (**b**) Raman spectra of MoS_2_ in different regions of the same sample. The inset displays the relation between the frequency difference of the two peaks and the number of layers of MoS_2_.

Prior to the sulfuration of the MoO_3_ crystals, they were reduced by heating them at 300 °C for 24 h in a tube furnace in forming gas atmosphere, Figure 1b. This process yields MoO_3−x_ crystals. We found that this step is crucial to avoid the evaporation of MoO_3_ during the sulfuration process as MoO_3_ is a highly volatile material. In contrast, MoO_2_ is a more stable oxide [29]; in fact, by partially reducing the molybdenum trioxide, we observed improved stability of the material upon temperature increase. The MoO_3−x_ layers were then converted to MoS_2_ by a sulfuration process in a closed glass ampoule. The sample containing the MoO_3−x_ layers was sealed with sulphur powder at 10^−5^ mbar pressure. The ampoule was placed in a furnace at 500 °C for 5 h and then the temperature was increased at 600 °C for another 5 h. Once the sulfuration process was concluded, the temperature was slowly lowered to room temperature (Figure 1c). The number of MoS_2_ layers that we obtain depends on the starting MoO_3_ thickness. Therefore, with this method, we are able to obtain MoS_2_ continuous layers covering most of the mica substrate with regions of up to 300 × 300 µm^2^ with < 5 layers in thickness; an example is shown in Figure 2a. As discussed below, single-layer MoS_2_ could also be observed (see the discussion related to the scanning transmission electron microscopy results). It is important to note that when we tried to sulfurize the as-grown MoO_3_ layers, without the reduction step, we obtained thick MoS_2_ crystallites randomly deposited on both the ampoule surface and on the substrate. Scanning transmission electron microscopy (STEM) data was acquired in an aberration-corrected JEOL JEM-ARM200cF electron microscope (JEOL, Tokyo, Japan) operated at 80 kV.

## 3. Results and Discussion

### 3.1. Raman Characterization

In Figure 2a, we show an optical image of a thin and large-area MoS_2_ film on mica. We employed Raman spectroscopy to characterize the MoS_2_ film as this technique has been demonstrated to be a very powerful tool to characterize 2D materials [30,31]. Figure 2b presents the Raman spectra acquired on two locations (indicated in the figure) of the MoS_2_ film shown in Figure 2a. The characteristic E^1^_2g_ and A_1g_ phonon modes of MoS_2_ (around 380 and 415 cm^−1^) are clearly visible in the spectra [24,32]. One can determine the number of layers from the frequency difference between these two Raman modes. In the inset in Figure 2b, we show the relation between this frequency difference and the number of layers of MoS_2_, obtained from the literature [33,34], and we compare these values with those obtained in two spots in our sample to determine the number of layers, finding that the MoS_2_ specimen is composed of a bilayer and a four-layer region. We refer the reader to the Supplementary Materials for a Raman map of another thin MoS_2_ region.

### 3.2. XRD Characterization

**Figure 3:**
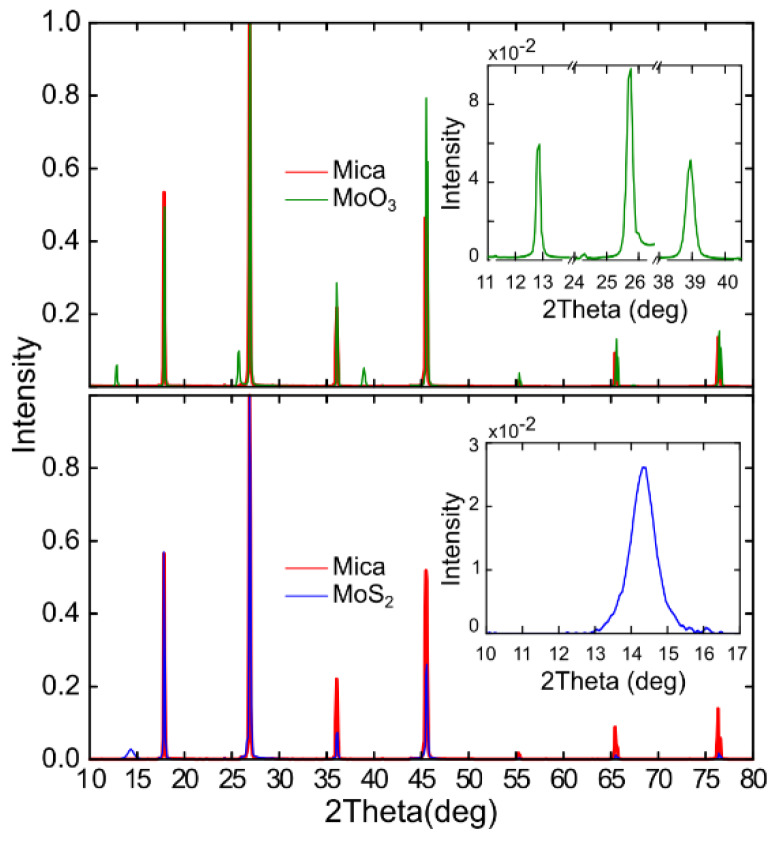
Comparison of XRD spectra of the sample shown in Figure 2, at different steps: MoO_3_ (initial) and MoS_2_ (after sulfuration) in green and blue, respectively. XRD spectra of a mica substrate in red to distinguish it from the peaks of the layer analyzed (insets).

The crystal structure of the films has been characterized with X-ray diffraction (XRD). XRD was performed at room temperature on the initial sample (MoO_3_ grown on mica 18 mm × 2 mm substrate) and on the final sample (MoS_2_ obtained after the sulfuration process). Figure 3 illustrates the X-ray diffractograms that were taken for the initial sample and for the final sample in green and blue, respectively. In red, we also show the X-ray diffractogram for a bare mica substrate, in order to differentiate the peaks that belong to the substrate from the peaks that correspond to the growth film.

Notice that the green spectrum exhibits peaks that correspond to (020), (040) and (060) reflections, which belong to the diffraction peaks of MoO_3_. The appearance of the (0k0) peaks, parallel to the plane (010), is a product of the preferred orientation of the MoO_3_ crystal with respect to the mica (001) surface due to the van der Waals epitaxy type of growth [35]. The blue spectrum obtained for the same sample after the sulfuration process shows a peak that corresponds to the (002) reflection of MoS_2_ [6]. Thus, we further confirm that we are able to obtain MoS_2_ from MoO_3_ deposited onto a mica substrate. In some works, it is proposed that the average thickness of a thin sample can be obtained from the analysis of the XRD peaks using the Scherrer equation (*D* = *kλ/β*cos*θ*, where *k* is the shape factor, *λ* is the X-ray wavelength, *β* is the full width at half maximum of the peak and 2*θ* is the scattering angle) [36,37]. By analyzing the (002) peak of the MoS_2_ XRD pattern, we estimated a c-stacking height for the analyzed sample of 10 nm, which corresponds to 15 layers of MoS_2_. Note that this value corresponds to the average thickness of the whole sample; however, thinner regions (such as those shown in Figure 2) can be found on it. It is also worth mentioning that the single-crystal domain size observed in our samples is also of the order of ~10 nm (see STEM discussion below) and thus it is not completely clear if the Scherrer equation provides accurate values of the average thickness of the sample or simply the single-crystal domain size.

### 3.3. STEM Characterization

**Figure 4:**
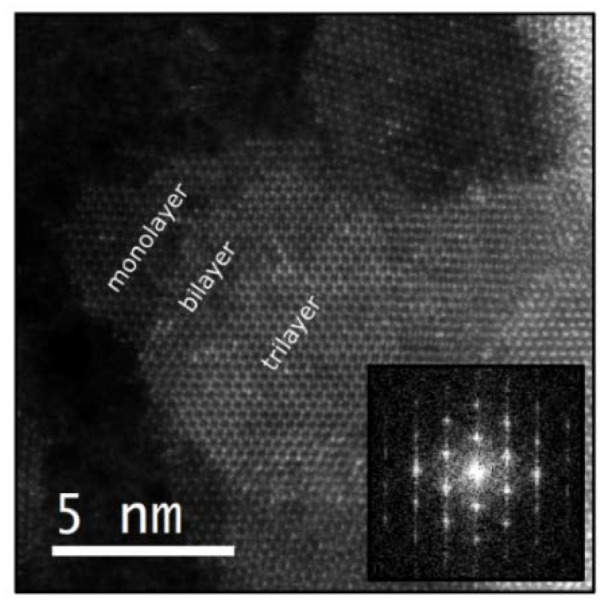
High-magnification HAADF images of a MoS_2_ thin film transferred over a holey Si_3_N_4_ membrane support. Inset shows FFT where a clearly hexagonal symmetry is exhibited.

The crystal structure of the films can be further characterized in real space by STEM. Figure 4 displays a high-angle annular dark field (HAADF) image of a MoS_2_ layer transferred over a holey Si_3_N_4_ membrane support by an all-dry deterministic transfer process [38]. In order to transfer the MoS_2_ films on mica, we stuck a polydimethylsiloxane (PDMS) sheet on its surface and we immersed it in distilled water. Due to the hydrophilic character of mica, the water wedges between de MoS_2_ and the mica surface, separating the MoS_2_ layer, which remains attached to the PDMS substrate, from the mica surface. The MoS_2_ is easily transferred to the membrane by gently pressing the PDMS containing the MoS_2_ film against the acceptor substrate and peeling it off slowly.

The STEM characterization indicates that the MoS_2_ film is polycrystalline, with a single-crystal domain size of 10–20 nm. Thinner regions can be found at the edges of the sulfurized film, where one can find monolayer, bilayer and trilayer areas (Figure 4 shows the edge of an MoS_2_ film, where mono-, bi- and tri-layer areas can be resolved). The fast Fourier transform (FFT) obtained from the monolayer region clearly shows the hexagonal symmetry of MoS_2_.

### 3.4. Electrical Characterization

**Figure 5:**
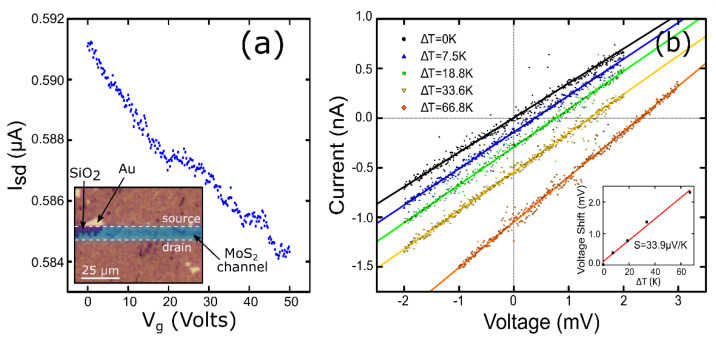
(**a**) Source-drain current vs. gate voltage measured in dark conditions and at *V*_sd_ = 1 V. The inset shows an optical image of the device measured (channel length = 10 µm, channel width = 1 mm). (**b**) Seebeck effect measurement on an MoS_2_ layer on a mica substrate by applying a temperature difference between electrodes. The inset shows the linear relationship between the thermovoltage shift and the difference in temperature. The Seebeck coefficient can be readily extracted from the slope.

The electrical properties of the fabricated MoS_2_ films were characterized by fabricating a field-effect device, by transferring a MoS_2_ film onto a SiO_2_/Si with pre-patterned drain-source electrodes separated by 10 µm. Figure 5a shows the measured source-drain current vs. gate voltage (*I*_sd_-*V*_g_) characteristics for a fixed source-drain voltage of *V*_sd_ = 1 V. Surprisingly, we obtained a decrease in the source-drain current upon gate voltage increase without reaching the OFF state, which corresponds to a strong p-doped field effect behavior. To confirm this fact, we performed a thermopower measurement. Figure 5b displays the *IV* characteristics acquired, applying a temperature difference between the two electrodes. It can be seen that a positive voltage offset at zero current appears (thermoelectric voltage) when the temperature different increases. The inset shows the thermoelectric voltage versus the temperature difference. The Seebeck coefficient can be extracted from the slope of a linear fit to the data: *S* = +33.9 μV/K. This positive value confirms the p-doped nature of the MoS_2_ film obtained by the direct sulfurization of crystalline MoO_3_. The low magnitude of the Seebeck coefficient also indicates a high doping level. We have carried out preliminary Hall effect measurements by backing up the p-type electrical behavior of the MoS_2_ films observed in the Seebeck and electric-field measurements. Unfortunately, the large resistance of our samples precludes us from quantifying the charge carrier concentration as the electronics of our Hall effect measuring system are optimized for low-impedance samples. The highly linear shape of the *IV*s, together with the high doping inferred from the shallow transconductance and low Seebeck coefficient, points to an Ohmic contact in the Au-MoS_2_ junction. We also estimated the resistivity of the device as ~100 Ω·cm, which is significantly higher than that of single-crystal MoS_2_ (~1–5 Ω·cm), [39,40] as expected from the small single-crystal domain size of our synthetic MoS_2_ layers.

In order to get a deeper insight into the microscopic origin of this p-doping in our MoS_2_ layers, we have done an electron energy loss spectra (EELS) analysis of the STEM data (see Supplementary Materials). Apart from the presence of Mo and S, we found C (which could come from e-beam-induced deposition of amorphous carbon during the STEM measurement), O and B. The presence of O could be due to an incomplete MoO_3_-to-MoS_2_ transformation, and the presence of B impurities could come from unintentional cross-contamination from the surface of the glass ampoules used during the growth. The presence of these foreign species could be a plausible source of the unexpected p-type doping.

**Figure 6:**
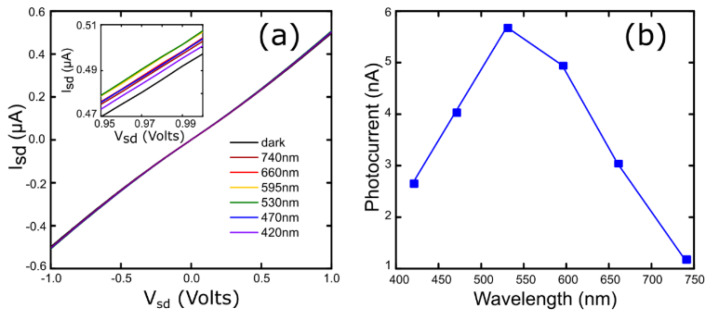
(**a**) *I*_sd_*-V*_sd_ curves for different illumination wavelengths and *V*_g_ = 0 V. Inset shows a smaller range to facilitate the visualization of the generated photocurrent. (**b**) Photocurrent spectrum obtained from *I*_sd_*-V*_sd_ curves.

Figure 6a represents the measured *I*_sd_-*V*_sd_ characteristics in dark conditions and under light excitation with different wavelengths. The gate voltage was set to *V*_g_ = 0 V during the measurement. Fiber-coupled LED light sources were employed to illuminate the device. The inset of this figure zooms in on the high voltage region of the traces to distinguish the differences induced upon illumination. The photocurrent as a function of the wavelength can be calculated from these data, as we show in Figure 6b. This spectrum reveals that the maximum photocurrent value is located between 530 and 595 nm, whereas it decreases at longer wavelengths. We were not able to measure a sizeable photocurrent beyond 740 nm, as expected for multilayer MoS_2_.

## 4. Conclusions

In summary, we presented an alternative method to obtain atomically thin MoS_2_ layers through the direct transformation of crystalline molybdenum trioxide (MoO_3_) layers into MoS_2_ nanosheets by sulfurization. The process can be carried out at moderate temperatures and using simple instrumentation. We obtained large-area polycrystalline MoS_2_ sheets two to four layers thick and we characterized them by Raman spectroscopy, X-ray diffraction and transmission electron microscopy. Regarding their electronic properties, they are strongly p-doped.

## Supplementary Materials

The following are available online at https://www.mdpi.com/1996-1944/13/10/2293/s1, Figure S1: (a) Raman map showing the difference between the E^1^_2g_ and A_1g_ peaks. The map shows a large region of ~4 layers, according to the peak difference value, and a thicker region in the bottom left corner. (b) Optical image of the same region studied in (a), Figure S2: (a) High magnification HAADF image of a MoS_2_ thin film transferred over a holey Si_3_N_4_ membrane support. (b) Electron energy loss spectra (EELS) acquired while scanning over the area in (a) for a total time of 20 s.
