# Supplementary material for: Direct Transformation of Crystalline MoO3 into Few-Layers MoS2
Source: Materials (Basel). 2020 May 15;13(10):2293. doi: 10.3390/ma13102293 (PMC7287652; doi:10.3390/ma13102293)
Supplement: Supplementary file 1 [file materials-13-02293-s001.pdf]

materials

MDPI

Supplementary Materials

Direct Transformation of Crystalline MoO_3 into Few-Layers MoS_2

Felix Carrascoso [1,*], Gabriel Sánchez-Santolino [1,3], Chun-wei Hsu [1,2], Norbert M. Nemes [3], Almudena Torres-Pardo [4], Patricia Gant [1], Federico J. Mompeán [1], Kourosh Kalantar-zadeh [5], José A. Alonso [1], Mar García-Hernández [1], Riccardo Frisenda [1] and Andres Castellanos-Gomez [1,*]

[1] Materials Science Factory, Instituto de Ciencia de Materiales de Madrid (ICMM-CSIC), E-28049 Madrid, Spain; gsanchezsantolino@ucm.es (G.S.-S.); chunwei.hsu@mail.mcgill.ca (C.-w.H.); patricia.gant@csic.es (P.G.); federico.mompean@csic.es (F.J.M.); ja.alonso@icmm.csic.es (J.A.A.); marmar@icmm.csic.es (M.G.-H.); riccardo.frisenda@csic.es (R.F.)

[2] Kavli Institute of Nanoscience, Delft University of Technology, Delft 2600 GA, The Netherlands.

[3] Departamento de Física de Materiales, Universidad Complutense de Madrid, E-28040 Madrid, Spain; nmnemes@fis.ucm.es

[4] Departamento de Química Inorgánica, Facultad de Químicas, Universidad Complutense, E-28040 Madrid, Spain; atorresp@ucm.es

[5] School of Chemical Engineering, University of New South Wales, Kensington, NSW 2052, Australia; k.kalantar-zadeh@unsw.edu.au

* Correspondence: felix.c@csic.es (F.C.); andres.castellanos@csic.es (A.C.-G.)

Received: 17 April 2020; Accepted: 13 May 2020; Published: date

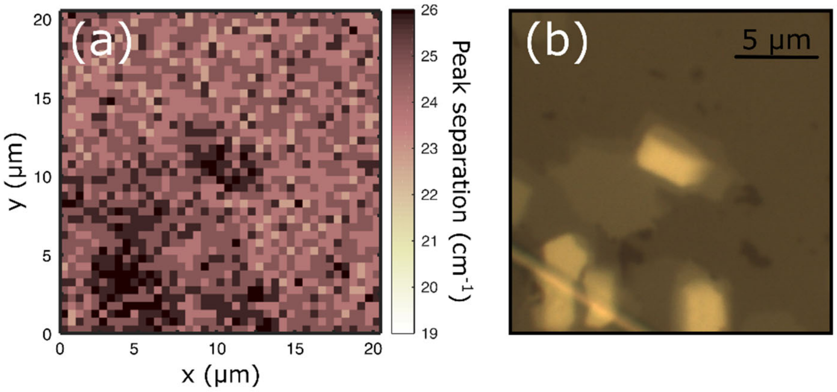

Figure S1. (**a**) Raman map showing the difference between the E^1_{2g} and A_{1g} peaks. The map shows a large region of ~4 layers, according to the peak difference value, and a thicker region in the bottom left corner. (**b**) Optical image of the same region studied in (**a**).

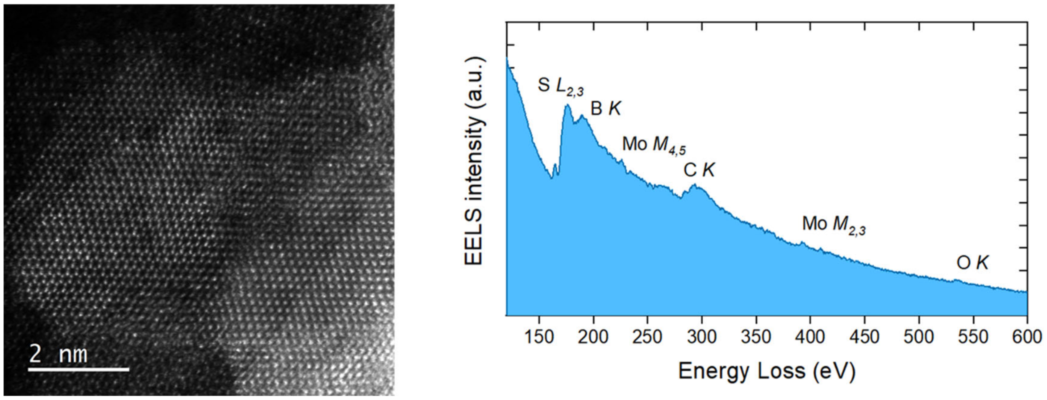

Figure 2. (**a**) High magnification HAADF image of a MoS_2 thin film transferred over a holey Si_3N_4 membrane support. (**b**) Electron energy loss spectra (EELS) acquired while scanning over the area in (**a**) for a total time of 20 s.

© 2020 by the authors. Submitted for possible open access publication under the terms and conditions of the Creative Commons Attribution (CC BY) license (http://creativecommons.org/licenses/by/4.0/).
